# Human Telomeres Are Hypersensitive to UV-Induced DNA Damage and Refractory to Repair

**DOI:** 10.1371/journal.pgen.1000926

**Published:** 2010-04-29

**Authors:** Patrick J. Rochette, Douglas E. Brash

**Affiliations:** 1 Department of Therapeutic Radiology, Yale School of Medicine, New Haven, Connecticut, United States of America; 2 Yale Comprehensive Cancer Center, Yale School of Medicine, New Haven, Connecticut, United States of America; 3 Department of Genetics, Yale School of Medicine, New Haven, Connecticut, United States of America; 4 Department of Dermatology, Yale School of Medicine, New Haven, Connecticut, United States of America; Stanford University School of Medicine, United States of America

## Abstract

Telomeric repeats preserve genome integrity by stabilizing chromosomes, a function that appears to be important for both cancer and aging. In view of this critical role in genomic integrity, the telomere's own integrity should be of paramount importance to the cell. Ultraviolet light (UV), the preeminent risk factor in skin cancer development, induces mainly cyclobutane pyrimidine dimers (CPD) which are both mutagenic and lethal. The human telomeric repeat unit (5′TTAGGG/CCCTAA3′) is nearly optimal for acquiring UV-induced CPD, which form at dipyrimidine sites. We developed a ChIP–based technique, immunoprecipitation of DNA damage (IPoD), to simultaneously study DNA damage and repair in the telomere and in the coding regions of *p53*, 28S rDNA, and mitochondrial DNA. We find that human telomeres *in vivo* are 7-fold hypersensitive to UV-induced DNA damage. In double-stranded oligonucleotides, this hypersensitivity is a property of both telomeric and non-telomeric repeats; in a series of telomeric repeat oligonucleotides, a phase change conferring UV-sensitivity occurs above 4 repeats. Furthermore, CPD removal in the telomere is almost absent, matching the rate in mitochondria known to lack nucleotide excision repair. Cells containing persistent high levels of telomeric CPDs nevertheless proliferate, and chronic UV irradiation of cells does not accelerate telomere shortening. Telomeres are therefore unique in at least three respects: their biophysical UV sensitivity, their prevention of excision repair, and their tolerance of unrepaired lesions. Utilizing a lesion-tolerance strategy rather than repair would prevent double-strand breaks at closely-opposed excision repair sites on opposite strands of a damage-hypersensitive repeat.

## Author Summary

Telomeres consist of a repeated sequence located at each end of each chromosome. This repeated sequence is required for chromosomal stability and integrity, a function important for both cancer and aging. The DNA sequence of human telomeres is 5–10 kb of a repeated double-strand hexamer (5′TTAGGG/5′CCCTAA). In theory, this sequence is nearly optimal for acquiring UV-induced DNA damage. We developed a novel technique, the immunoprecipitation of DNA damage (IPoD), to study DNA damage induction and repair in the telomere and in coding regions (*p53*, 28S rDNA, and mitochondrial DNA). We find that human telomeres are hypersensitive to UV-induced DNA photoproducts and that the removal of those DNA photoproducts is almost absent. Cells containing persistent high levels of telomeric DNA damage nevertheless proliferate and chronic UV irradiation of cells does not accelerate telomere shortening. Telomeres are therefore unique in at least three respects: their biophysical UV sensitivity, their prevention of excision repair, and their tolerance of unrepaired lesions.

## Introduction

Telomeric DNA consists, in all eukaryotes examined to date, of a tandemly repeated sequence located at each end of each chromosome. In humans, it is constituted of 5–10 kb of a repeated hexamer (5′TTAGGG/5′CCCTAA). Telomeres are required for chromosomal stability and integrity (reviewed in [1]).

Telomeres are hypersensitive to single-strand DNA damage induced by oxidative stress. This is thought to be due to the fact that sequences containing guanine triplets are highly sensitive to oxidation [2], [3]. When inserted in a plasmid, telomere sequence is 7-fold more sensitive to Fe2+/H_2_O_2_-induced strand breakage than bulk sequence [2]. Moreover, breaks induced in telomeres are repaired significantly more slowly than in other sequences, including interstitial guanine rich repetitive sequence tracts; repair is still incomplete after 19 days compared to complete repair at 1 day elsewhere [4]. In addition, the oxidation of telomeric DNA contributes to their premature shortening. The frequency of oxidative DNA damage at the telomere correlates with the amount of telomere lost during subsequent rounds of DNA replication [5]. It was proposed that the telomere enters DNA replication with greater oxidative DNA damage than the rest of the genome and this elevated damage contributes to telomere shortening [6]. Contrasting with this hypothesis, however, it has been shown that telomere shortening induced by oxidative DNA damage can be replication independent [3].

Ultraviolet light-induced DNA damage has been used for decades as a model to study DNA damage induction and repair. It is biologically relevant because UV is a complete carcinogen, requiring no additional treatments for tumor development, and is the preeminent risk factor in skin cancer development. The vast majority (>80%) of UV-induced damage in B-form DNA consists of cyclobutane pyrimidine dimers (CPD) [7], [8]. CPDs are intra-strand DNA lesions formed when two adjacent pyrimidines are joined across their 5–6 double bonds due to UV-excitation of one of them. The most frequent is the TT cyclobutane dimer [9]. These photoproducts are repaired by the nucleotide excision repair (NER) pathway, which nicks the DNA backbone and excises the damaged segment. Theoretically, the telomere sequence constitutes a perfect target for UV-induced DNA damage. First, the TT on the G-rich strand is repeated thousands of times in each chromosome. On the other strand, the 5′CCCTAA3′ would nominally generate low frequency CC and CT CPD, but two factors supervene: tracts of adjacent pyrimidines tend to generate multiple CPDs on the same molecule, due to cooperative denaturation of the helix by each successive CPD [10] and A:T tracts tend to transfer energy down the base stack until depositing it at a G:C pair [11], [12]. These potential CCCT dimer tracts are again repeated thousands of times in each chromosome. These considerations suggested that this sequence might constitute a hotspot for UV-induced damage. The presence of potential hotspots on both telomeric strands then raises the following spectre: if the cell attempts to simultaneously repair two nearby CPDs on opposite strands, the twin incision nicks would mimic a double-strand DNA break [13]–[15], triggering a DNA damage response and chromosome aberrations [16], [17].

Studying DNA damage induction and repair in the telomere is challenging. The vast majority of the techniques used to study DNA damage induction and repair in a specific part of the genome are PCR based [18]. Because telomeres are constituted of repeated sequences, there are no unique PCR-primer sites. Mismatch primers have been developed to analyze human telomere length by quantitative PCR [19]. However, since those primers can bind to any repeat element of the telomere sequence, they cannot be used in standard techniques to study DNA damage induction and repair, which rely on having one or two known DNA ends. An older study used a single-enzyme modification of the telomere restriction fragment technique (TRF) to study UV-induced CPD in telomeres [20]. However, it is now known that the TRF technique does not provide information on the true length of telomeres [21]: restriction enzymes used to cleave non-telomeric DNA (*HinfI* or *RsaI*) give TRF lengths that depend on the site of restriction in the pre-telomeric region. The situation is exacerbated by the fact that achieving complete digestion of genomic DNA using a single restriction enzyme is challenging. Thus studying the induction of DNA damage using the TRF technique does not provide information exclusively about telomeres but about a mixture of telomeric and pre-telomeric DNA. Pre-telomeric DNA is now known to be one of the most rapidly-repaired regions of the genome [22], skewing lesion measurements if this region is included.

We developed a novel method, based on the chromatin immunoprecipitation technique (ChIP), to study single-strand DNA damage. This technique, “immunoprecipitation of DNA damage” (IPoD), allows the separation of damaged DNA from undamaged. The result is two fractions that can each be quantitated by PCR using primers specific for the gene under study. Previously developed primers specific for the human telomeric sequence [19] can be used in this technique, allowing the study of single-strand DNA damage induction and repair in this region. Using the IPoD technique, we have studied UV-induced CPD induction and repair in the telomere as well as in the *p53* tumor suppressor gene, in 28S ribosomal DNA, and in a portion of mitochondrial DNA. We find that the telomere sequence is highly sensitive to the induction of CPD by UV light. Moreover, we show that the repair of those UV-induced CPD in telomeres is nearly absent.

## Results

### Immunoprecipitation of DNA Damage (IPoD) Is a Quantitative Technique

**Figure 1:**
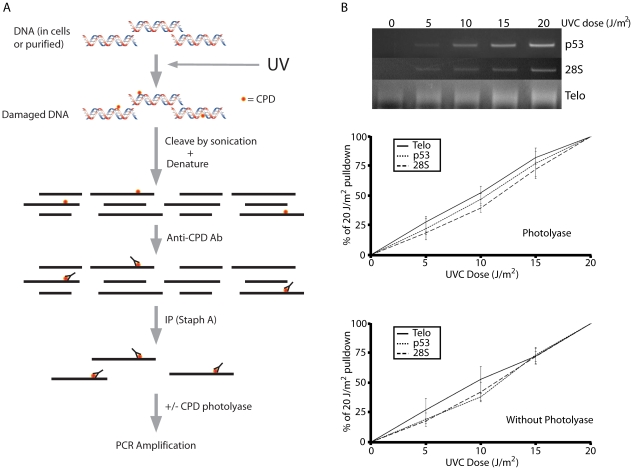
The ImmunoPrecipitation of DNA Damage technique (IPoD). (A) Schematic representation of the technique. Damaged or control DNA is sonicated to 500–1,000 bp fragments, denatured, and immunoprecipitated (IPd) using a DNA lesion-specific antibody and staph A beads. (B) Linearity of the IPoD signal at minimally-lethal doses. UV-induced cyclobutane pyrimidine dimers (CPD) in human diploid fibroblasts were assayed at low UV doses in the *p53* gene, the 28S ribosomal DNA gene, and the telomeres. For each sample, the integrated intensity of the band or lane containing the PCR–amplified IP pulldown fraction is normalized against the unamplified input DNA for that sample. For comparison between doses, the pulldown percentages at 0 and 20 J/m^2^ were assigned a value of 0% and 100%, respectively. The dose response is linear for each genome region analyzed and this linearity was not affected by removing CPD before PCR using photolyase. Each result depicted in (B) is derived from triplicate experiments.

IPoD is based on the ChIP technique [23]. Instead of immunoprecipitating a protein covalently cross-linked to DNA, IPoD directly immunoprecipitates DNA fragments containing a DNA structural alteration. Here we use the IPoD technique to study the CPD damage induced on a DNA strand by UV radiation [9]. The technique is schematized in Figure 1A. As the level of DNA damage in a specific region of the genome increases, the number of immunoprecipitated fragments from this region will increase. UV-irradiated DNA, but not unirradiated DNA, yielded an IP fraction using antibody against CPD but not with antibody to Bcl-xL protein or with antibody omitted (Figure S1). UVC has been used in this study to minimize the introduction of photosensitized oxidative DNA damage that accompanies UVB.

The quantity of specific genomic DNA fragments present in the IP fraction was measured, after removing CPDs using photolyase, by PCR amplification using primers specific for the *p53* tumor suppressor gene, the 28S ribosomal RNA repeat region, the *CYTB* gene of mitochondrial DNA, and telomeric DNA. The telomere sequence is composed of a 6-mer concatenated to greater than 5 kb, complicating the design of PCR amplification primers. A 5′ 21-mer primer composed of telomeric repeats is certain to have a complement on the 3′ primer, so primers will anneal together instead of annealing to the telomeric DNA target. Cawthon [19] describes telomeric primers containing mismatches that prevent primers from annealing to each other, thus achieving preferential annealing to telomeric DNA. Because the particular site at which any primer binds on the telomere sequence is random, the resulting PCR product is not a sharp band but a smear.

For the exponential PCR process to be used quantitatively, it must contain an internal control, as in real-time PCR, or be carried out so that all samples have been amplified by the same factor of 2^n^, that is, with all samples lying on the log-linear part of the amplification curve so that they can be compared to a calibration curve. No internal control is possible with IP, so we adjusted the amount of starting DNA material and the number of PCR cycles to achieve log-linearity for each primer. Figure 1B (upper two panels) shows that the signal from the PCR amplified IPoD-immunoprecipitated DNA is proportional to the UVC dose for 3 different genomic regions. Each genomic region's signal is normalized to that region's signal at 20 J/m^2^. The signal was linear up to 30 J/m^2^ UVC (Figure S2). Above this dose range, the slope decreased. Doses above 20 J/m^2^ UVC are lethal so the present experiments did not enter that range. The high-dose slope reduction could be due to sustaining more than 1 CPD per DNA fragment, saturating the anti-CPD antibody with CPDs, or depleting PCR reagents. Linearity at doses below 30 J/m^2^ UVC indicates that: a) CPDs are not missed because they occur in DNA segments that will already be IPd due to another CPD; b) the many telomere copies do not saturate the PCR reaction; and c) CPDs or (6-4) photoproducts remaining in the fragment during PCR do not cause a dose-dependent dropout of sample.

To confirm the last point, we also amplified the IP fraction without first reversing remaining CPD with photolyase (Figure 1B, lower panel). When normalized to the signal at 20 J/m^2^, the shape and slope of the dose-response curve were unchanged for both single-copy and repeat genes. Because PCR-blockage is sometimes used as a relatively insensitive lesion assay, this might seem paradoxical. But the goal of the PCR blockage assay is to determine whether the extent of amplification is reduced compared to undamaged DNA, by measuring the percentage of fragments that have no lesions between the PCR primers. In contrast, IPoD has already identified the CPD-containing fragments via the IP step, so the CPDs can lie outside the PCRd region. The IPoD amplification serves only to make visible a particular set of CPD-containing fragments present in the IP sample. Even when some photoproducts are present, as in the absence of photolyase, the signal is nearly normal: a) 60% of the ∼750 bp sheared fragment lies outside the ∼300 bp PCR fragment; thus, even if photoproducts are a complete block to PCR, the PCR primers are assaying a CPD-target region *external* to the PCR primers rather than internal plus external. b) Diminution of a gene's PCR signal due to a photoproduct internal to the primers is equal between genes, on average, because every IPd molecule has by definition at least one cyclobutane dimer and, at the UV doses used, typically no more than one dimer per molecule. c) PCR inhibition is only partial because i) Taq polymerase can slowly bypass CPDs [24] and ii) partially-extended fragments will, in the next PCR cycle, anneal to a different partner and extend further; thus the internal region is sampled as well.

### Telomeres Are Hypersensitive to UV-Induced DNA Damage

To compare the level of UV-induced CPDs in telomeres with the level in other genomic regions, we calculated for each region the absolute percentage of the input that was IPd (IP/Input). This absolute number circumvents differences in PCR efficiency or copy number. The IP fraction was amplified using primers for *p53*, 28S rDNA, and the telomere after removing CPD with photolyase. For the corresponding Input DNA, various dilutions were amplified and a calibration curve of PCR signal vs dilution was constructed. The PCR signal from the IP was compared with the curve to determine the dilution factor matching the IP signal, and thus the ratio IP/Input.

**Figure 2:**
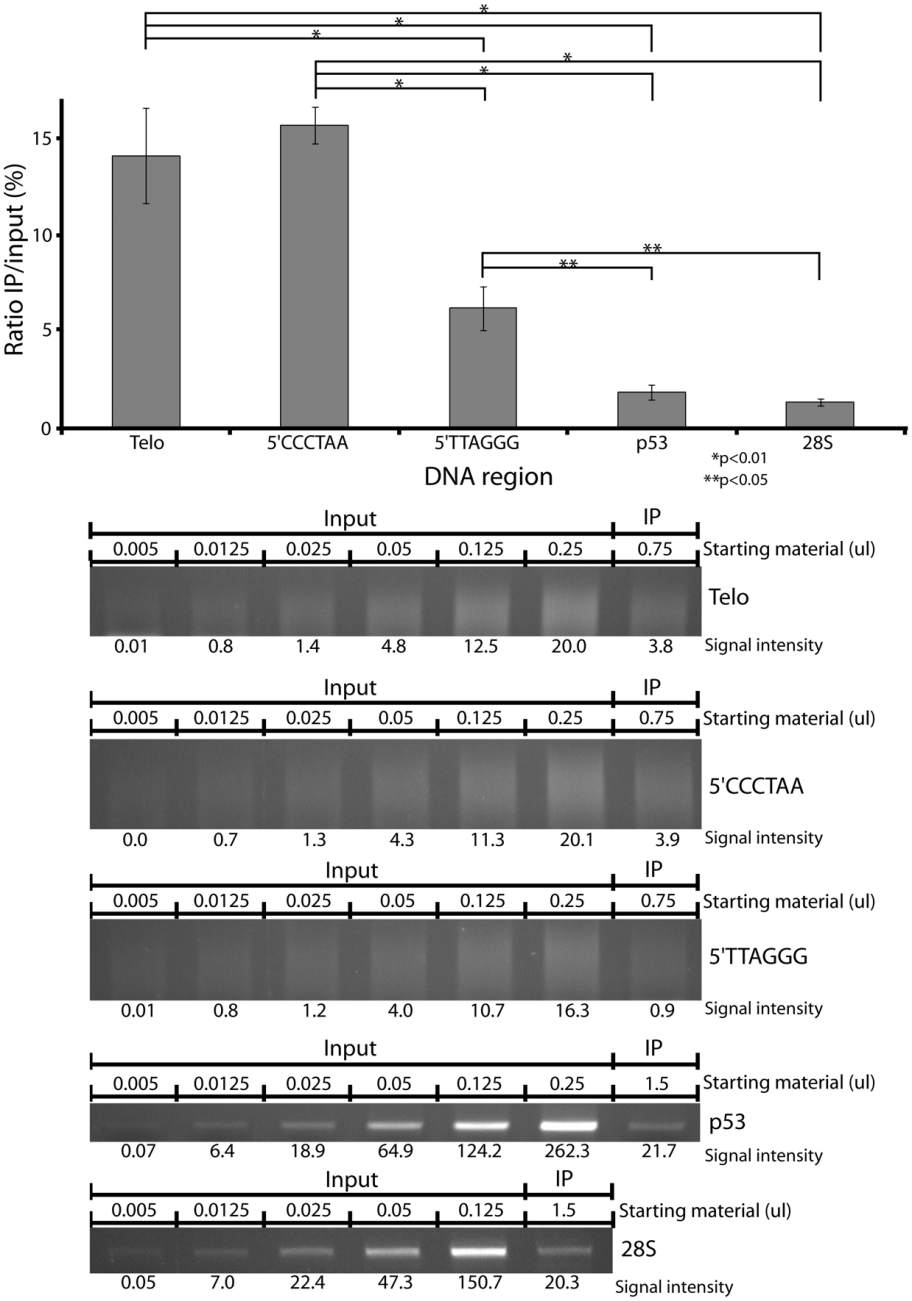
Hypersensitivity of telomeres relative to coding regions. The frequency of CPD is ∼7 fold higher in human fibroblast telomeres than in a fragment of the *p53* gene or 28S ribosomal DNA at 20 J/m^2^ UVC. This sensitivity also holds for each telomere strand individually and is unaffected by precise correction for the percentage of dimerizable dipyrimidines in the various sequences. (Upper) Graphical representation of the quantification of the PCR–amplified bands or lanes. The ratio IP/input corrects for copy number. (Lower) Gels of PCR–amplified IP DNA. Each experiment was performed in triplicate. P values are derived from the two-tailed heteroscadastic Student's t-test.

At 20 J/m^2^, 14% of the telomeric DNA fragments were damaged (Figure 2), whereas approximately 2% of fragments from the *p53* or 28S rDNA genes were damaged at the same dose. The same ratios were obtained whether or not remaining CPD were reversed with photolyase prior to PCR amplification (not shown). Therefore, the telomeric region is 7 times more sensitive than two other regions of the genome. To determine whether one of the telomeric DNA strands was responsible for this sensitivity, we examined the strands separately. Because each strand of the telomere contains only 3 of the 4 possible nucleotides (only GAT for the 5′TTAGGG strand and only ATC for the 5′CCCTAA strand), we performed a strand-specific amplification of the telomere by omitting one nucleotide from the reaction. In addition, an initial linear amplification using only one of the 2 primers and 3 of the 4 nucleotides was performed for 30 cycles. Linear amplification was followed by a standard PCR amplification of the linear-amplified DNA (see Materials and Methods). Each strand was more sensitive than *p53* or rDNA (Figure 2), with 16% of the 5′CCCTAA strand fragments being damaged at 20 J/m^2^ UVC and 6% of the 5′TTAGGG strand.

The telomeric sensitivity was not due to a difference in the frequency of dipyrimidine sites (the site of formation of cyclobutane dimers). This frequency was 29.5 dipyrimidine sites per 100 nucleotide in the *p53* fragment, 28.9 in the rDNA fragment, and 33.3 in the telomere. We also examined possible artifactual explanations for the telomeric sensitivity. First, repeated DNA at the ends of chromosomes might sonicate differently, producing more-readily IPd fragments. A Southern blot showed that the sizes of sheared telomere and *p53* DNA are the same (Figure S3A). Second, telomeric DNA might have a conformation more accessible to antibody or enzymes. A similar Southern blot experiment revealed that photolyase could completely reverse cyclobutane dimers in both telomeres and *p53*, suggesting that, at least in naked DNA, accessibility differences do not play a role (Figure S3B). Thirdly, the large number of telomeric repeats might create shorter PCR fragments, which would PCR more efficiently. But the number-average molecular weight of the telomere smear is 250–500 bp, the same range as the ∼300 bp *p53* and 28S bands. Finally, we considered that more ‘copies’ of the (diluted) telomeric repeat are present in the PCR reaction than are *p53* copies, but this is also true for its pre-IP control.

### Repeatedness *Per Se* Renders Telomeres Sensitive to UV

**Figure 3:**
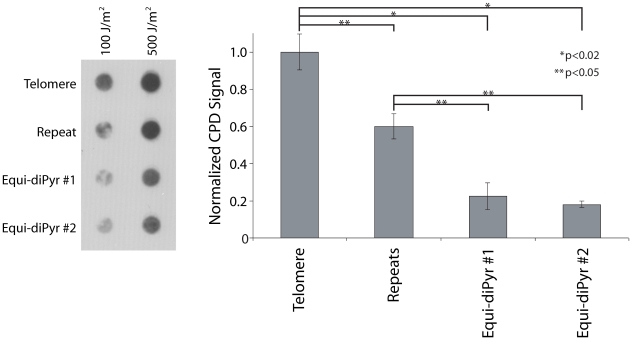
Repeatedness *per se* sensitizes telomeres to UV. 400 ng of a 102-mer double-stranded oligonucleotide containing either 10 telomere repeats (Telomere), 10 repeats of a non-telomeric sequence (5′TTCGGG) (Repeats) or a non-repeat region containing the same number of pyrimidine sites (Equi-diPyr #1 and Equi-diPyr #2) were irradiated with UVC (100 or 500 J/m^2^), applied to a dot-blot, and the CPD-containing DNA was revealed using an anti–CPD antibody. Quantification of the dot-blot is graphically represented on the right. The various oligonucleotide constructs were normalized to the telomeric repeat oligonucleotide: A value of 1 was assigned to the telomere CPD signal at 100 and 500 J/m^2^. The signal of the other conditions (repeat, Equi-diPyr #1 and #2) indicates the ratio between the intensity of the dot-blot at those conditions and the telomere. Because there was no dose-dependency in the signal ratio for any condition, it was then possible to average the signal ratios of 100 and 500 J/m^2^. The telomere repeat oligo was 5-fold more sensitive to UV than either oligo containing the same number of dipyrimidine sites but randomly distributed (non-repeated, Equi-diPyr). The oligo containing an arbitrary repeat was 3-fold more sensitive than the Equi-diPyr oligos. Each experiment was performed in triplicate. P values are derived from the two-tailed heteroscadastic Student's t-test.

To confirm the UV hypersensitivity of telomeres independently of IPoD, and to test whether the telomere's hypersensitivity was due to its DNA sequence independent of telomere-bound proteins such as shelterins or chromatin-induced DNA conformation, we examined synthetic oligonucleotides. Four different double-stranded 102-mer oligonucleotides were constructed in which the central 60 bp were varied to include either: 10 repeats of the telomere sequence (5′TTAGGG/CCCTAA) (“Telomere”), 10 repeats of a modified 6-mer (5′TTCAGG/CCTGAA) having the same number of potential UV photoproduct sites (dipyrimidine sites) (“Repeats”), or a single random sequence containing the same number of potential UV photoproduct sites (two examples, “Equi-diPyr #1” and “Equi-diPyr #2”). Each 102-mer was irradiated with either 100 or 500 J/m^2^ UVC (0.1 – 0.5 CPD per molecule). The irradiated double strand oligonucleotides were directly applied onto a nylon membrane (without PCR amplification) using a dot-blot apparatus and CPD-containing DNA was detected using a CPD-specific antibody (Figure 3). The quantification shows that the telomere repeat was 5 times more sensitive to UVC-induction of CPD than either of the non-repeated sequences. Surprisingly, the non-telomeric 6-mer repeat (5′TTCAGG) was 3 times more sensitive than the random (non-repeated) sequences. This result suggests that repeatedness *per se* renders dipyrimidine-containing oligonucleotides more sensitive to UV, with telomeric sequences being particularly sensitive.

### The Telomeric Sequence Acquires UV-Sensitivity above 4 Repeats

**Figure 4:**
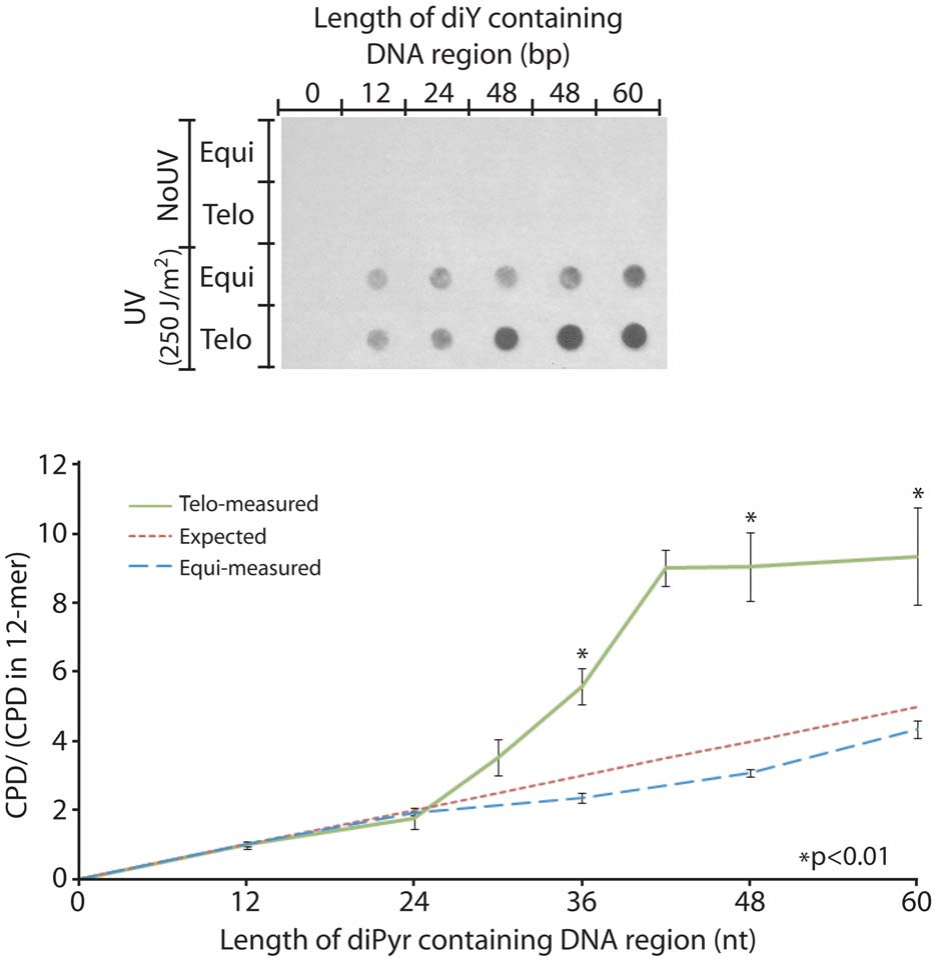
The telomeric sequence acquires UV sensitivity above 4 repeats. To determine the influence of repeat length on UV sensitivity, increasing numbers of telomeric repeats (double-stranded 6-mer, 5′TTAGGG3′ and its complement) were embedded in 102-mer double-strand oligonucleotides that contained no dipyrimidines outside the telomeric region. This series will exhibit a trivial increase in the UV response simply because the number of potential CPD target sites at dipyrimidines increases. This increase is linear with repeat number and is shown as the “Expected” line (red). Each member of the “Equi” series of oligos contains the same number of dipyrimidine sites as the corresponding telomeric oligo but randomly distributed. These Equi oligos had the expected behavior (blue line). The “Telo” oligo series contain 2–10 telomeric repeats (12–60 bp diPyr region, green line). A phase transition in UV sensitivity is visible between 4 and 7 telomere repeats. The Equi and Telo oligos are compared by normalizing to their response at the shortest diPyr region length, the 12-mer. Each experiment used 400 ng of oligos irradiated at 250 J/m^2^ UVC, about 0.5 CPD per oligo, and was performed in triplicate. P values are derived from the two-tailed heteroscadastic Student's t-test.

To determine the number of repeats needed to confer sensitivity to CPD formation, we designed 102-mer double-strand oligonucleotides having increasing numbers of telomeric repeats (“Telo” series). As control, oligonucleotides were designed to have a dipyrimidine-containing region of the same length as the corresponding telomeric repeats but not arranged as repeats (“Equi” series). Outside the repeated region or the corresponding dipyrimidine-containing region, the oligonucleotide lacks dipyrimidine sites. For the “Equi” series, increasing the length of the dipyrimidine-containing region linearly increased the number of CPDs induced, as expected (Figure 4). The Telo series behaved similarly to the Equi oligonucleotides up to 4 repeats. Strikingly, a positive effect of repeats on UV induction of CPD appeared around 5 repeats, as if the DNA had undergone a phase transition. The oligonucleotide containing 5 telomere repeats was 3 times more sensitive than the non-repeated oligo. At 7 repeats, a plateau was reached at which sensitivity to CPD formation was 4–5 times greater in the oligonucleotide containing repeats than in the non-repeated oligo. Limitations on synthesizing longer telomeric oligonucleotides prevented us from determining whether the UV-susceptibility of repeats continues to increase with repeat number – with the plateau merely reflecting the fact that double-strandedness is partially lost at DNA ends – or truly plateaus due to complete acquisition of an altered conformation.

### Telomeres Do Not Repair Their Cyclobutane Dimers

To examine photoproduct repair, sub-confluent human diploid lung fibroblasts (WI38) were irradiated with a minimally lethal dose of UVC (10 J/m^2^) and harvested at different time points 0–48 hours post-irradiation. Photoproduct-containing DNA was then isolated using IPoD and, after photoreversing CPD, amplified using primers specific for the telomere region (“Telomere”), mitochondrial DNA (“mtDNA”), the gene for the RNA component of ribosomal subunit 28S (“28S”), and tumor suppressor gene *p53* (“p53”). *p53* was used as a positive control for fast repair by the transcription-coupled NER system (TCNER) [25] because *p53* is actively transcribed in human cells, especially after a genotoxic stress such as UV irradiation. The repair rate observed here will reflect both DNA strands and thus will be an average of TCNER on the transcribed strand and slower global genomic NER (GGNER) on the non-transcribed strand. CPDs in the 28S gene of mammalian cells are known to be repaired only by GGNER and not by TCNER [26]–[28], so it serves as a positive control for normal GGNER. In contrast, NER proteins are not present in mitochondria and CPD are not repaired in mtDNA [29]–[31]; thus mtDNA serves a negative control for repair and would indicate any apparent photoproduct loss due to cell dilution during replication.

**Figure 5:**
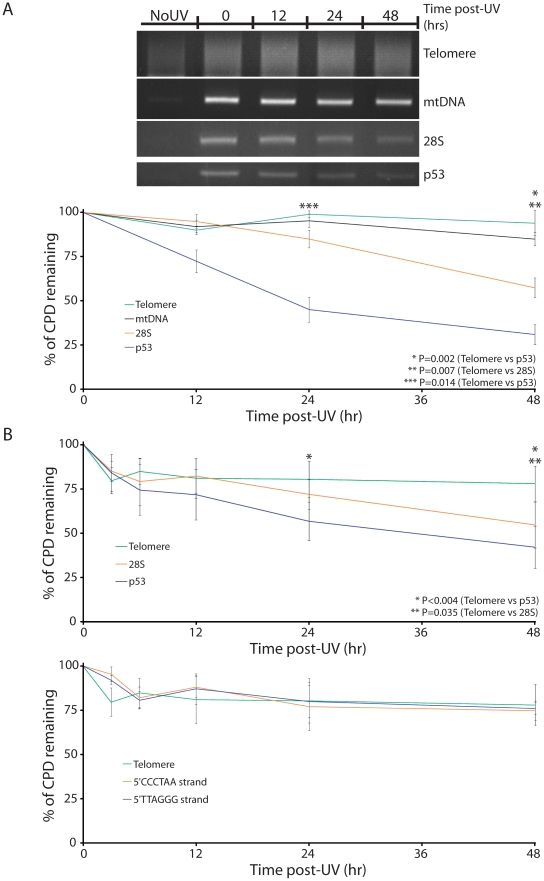
Absence of excision repair of CPDs in telomeres. (A) WI38 human diploid fibroblasts were irradiated with minimally-lethal doses of UVC (10 J/m^2^) and returned to the incubator for varying lengths of time before harvesting (0–48 h). The DNA was then isolated, the IPoD technique was used to isolate damaged DNA, photolyase was used to remove CPD, and PCR was performed on region of interest: Telomere, mitochondrial *CYTB* gene (mtDNA), 28S ribosomal DNA, or *p53*. For each time point, the integrated intensity of the band or lane containing the PCR–amplified IP pulldown fraction is normalized against the unamplified input DNA for that time point. The amount of DNA in the fraction pulled down by antibody to CPD decreases with time in the *p53* and 28S genes, reflecting normal excision repair, but not in telomeres or mtDNA (*CYTB* gene). (B) A similar experiment was performed in primary human skin fibroblasts UV-irradiated at 20 J/m^2^. In this experiment, CPD were removed from IPoD-immunoprecipitated CPD containing DNA using DNA photolyase before PCR amplification and repair in the two telomeric DNA strands was analyzed together and separately. Each experiment was performed in triplicate. P values are derived from the two-tailed heteroscadastic Student's t-test.

We found that, 48 hours post-UVC, approximately 70%, 40% and 10% of CPD were removed from *p53*, 28S and mtDNA DNA regions, respectively (Figure 5A). Repair of CPD in the telomere region was comparable to or less than that seen in the mtDNA negative control, less than 10% after 48 hr, indicating that the NER system is ineffective in telomeres. To ensure that the lack of repair in the telomere region was not specific to the cell line used, the growth stage, or the UV dose, the experiment was repeated in confluent (quiescent) skin fibroblasts at 20 J/m^2^ UVC, with the same result (Figure 5B).

### Telomeres Tolerate UV Photoproducts without Telomere Shortening

Cyclobutane pyrimidine dimers are profound blocks to DNA replication forks in mammalian cells, triggering cell cycle arrest and DNA damage responses through the ATR pathway [32], [33]. Oxidative damage at telomeres interferes with maintenance of the D loop and induces telomere shortening [3], [6], [34]. To determine how the elevated and persistent levels of CPD affect telomere maintenance, we investigated UV-induced telomere shortening.

**Figure 6:**
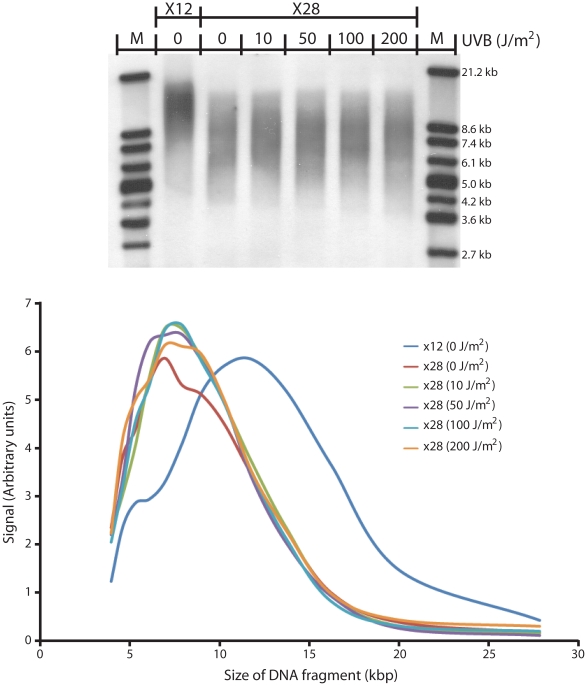
UV does not induce telomere shortening in replicating cells. Human diploid fibroblasts were irradiated between each passage (for 16 passages) with the indicated doses of UVB (0–200 J/m^2^). UVB induces ∼100 fold fewer CPDs per unit dose than UVC. After irradiating cells for 16 passages (to passage 28), DNA was harvested and the telomere restriction fragment (TRF) technique was used to determine telomere length. Digoxigenin-labeled marker (Roche Molecular Biochemicals, Indianapolis, IN) was used. Upper panel, agarose gel; lower panel, graph of band intensity at each size position. At passage 12, mean telomere length was ∼12 kb. At passage 28, the telomere length was ∼8 kb, independent of the UV dose used, corresponding to the expected telomere shortening with increasing passage level.

Human diploid fibroblasts were chronically irradiated with minimally-lethal doses of UVB, receiving 0 to 200 J/m^2^ UVB 1 day after each passage (approximately every 5 days). After 16 passages, DNA was isolated and approximate telomere length was measured using the telomere restriction fragment (TRF) technique [35]. At passage 12 (“X12”), the mean telomere length of un-irradiated cells was approximately 12 kb. At passage 28 (“X28”), the telomere length was approximately 8 kb (Figure 6), corresponding to the expected telomere shortening with increasing passage level. Irradiating cells with 10 to 200 J/m^2^ of UVB 16 times did not increase the rate of telomere shortening. Therefore, a) normal telomere shortening is not accelerated by unrepaired CPD and b) unrepaired CPDs are not removed by telomere shortening. Evidently, the telomere possesses an efficient tolerance mechanism for cyclobutane pyrimidine dimers.

## Discussion

Every eukaryote has telomeric DNA consisting of short sequences repeated thousands of times at the end of each chromosome. To these repeats has been attributed the role of preserving genome integrity via the stabilization of chromosomes. By “capping” chromosomes ends, telomeres protect them from recombination. A role of “longevity clock” has also been attributed to telomeres. Eukaryotes begin life with full-length telomeres and, at each cell division, telomeres shorten to finally reach a point where the cell enters into a senescence state. In view of these critical roles in genomic integrity, the telomere's own integrity should be of paramount importance to the cell. The present results show that telomeres are unique in at least three unexpected respects: their biophysical sensitivity, their prevention of repair, and their tolerance of unrepaired lesions.

### Biophysical Sensitivity of Telomeres to UV Light

Telomeres were found to be 7 times more sensitive to UV-induced CPD than other DNA regions (Figure 2). This observation was made in a cellular context, so the proteins and secondary structure of the chromatin might be involved in this hypersensitivity. To distinguish these possibilities, we tested the sensitivity of telomere sequence inserted in a 102-mer oligonucleotide. Because this oligonucleotide was irradiated *in vitro*, it was free of any cellular context. This oligonucleotide showed hypersensitivity comparable to the telomere DNA sequence *in vivo* (Figure 3). Thus the cellular context is not the major contributor to the UV hypersensitivity of the telomere. What, then, can explain it? We tested different oligonucleotides for UV sensitivity and found that short repeats, like the telomeric sequence, render those oligonucleotides more sensitive. An oligonucleotide containing 10×6-mer repeats was ∼5-times more sensitive than an oligonucleotide containing the same frequency of dipyrimidine sites but randomly distributed (not in repeats). Surprisingly, the sensitivity of the telomeric repeat underwent a sudden transition at 5 repeats, suggestive of a structural phase change (Figure 4). This result means that the expected sensitivity based on DNA sequence is not the entire source of UV sensitivity. The biophysical nature of this transition, and its effect on the distribution of DNA photoproducts, will require biophysical investigation. G-rich single strands undergo a variety of interactions such as Hoogsteen base pairing and G-G stacking. These can create G quadruplexes, parallel-stranded helixes, A- and Z-form DNA, hairpins, and local melting. In telomeric and trinucleotide repeats, the stability of the various structures depends on the number of repeats [36], [37]. The behavior in double-stranded DNA is less studied.

### Prevention of Repair

A region of the genome so critical to cell survival and genomic integrity would be expected to preserves its own integrity after a genotoxic stress. Yet little is known about how telomeric DNA does this. The finding that telomeres are hypersensitive to UV-induced DNA damage prompted the expectation that repair of this DNA damage would be rapid, to prevent DNA damage accumulation in this region. What we found was the contrary. Repair was almost absent in telomere regions, proceeding as slowly as in mitochondrial DNA where NER proteins are absent (Figure 5). Two days after UV irradiation, CPD were still present in telomeres but had been half removed from coding regions (*p53* or 28S genes) and probably entirely removed from the transcribed *p53* strand. The repair defect could be active or passive. In the passive category, compaction of telomeric heterochromatin may prevent access of repair proteins [38]. Also, telomeric DNA has been reported to have partial A-DNA character [39], which predisposes to *trans*- rather than *cis*-isomers of CPD [8]. Little is known about the repair of *trans*-isomers of CPD and they may be more difficult for the NER system to recognize or remove. In the active category, some of the many protein factors bound to telomeres (reviewed in [40]) may inhibit the repair system in this region.

There are two reasons suppression of excision repair can be desirable. The high frequency of CPDs in the telomere, together with the telomere's repeat nature, may generate multiply damaged sites (MDS). MDS are sites where DNA lesions are closer than ∼20 bp on opposite strands [41]. After the incision nicking that is the first step in excision repair, multiply damaged sites result in double-strand DNA breaks. This has been observed for UV-irradiated DNA containing halogenated nucleotide analogs in close proximity [41]. Double-strand breaks, in turn, are clastogenic and lethal events. At an MDS, displacement of the lesion-containing oligonucleotides during the second step of excision repair will also create overlapping daughter strand gaps [13]–[15]. This event increases the permissible distance between CPDs. In unique-sequence DNA, such MDSs would be rare, but in repeats they could be the rule when photoproduct frequency is high. The absence of telomere shortening after chronic UV irradiation (Figure 6) indicates that, in fact, such double-strand breaks have been avoided.

### Tolerance of Unrepaired Lesions

The fact that cell proliferation was unhindered by chronic UV irradiation, despite the presence of CPD in their telomeres, raises a new question: how can a cell tolerate DNA damage in its telomeres? During mammalian DNA replication, a bulky lesion such as a CPD typically blocks replication fork progression [42], [43]. This blockage leads to single-stranded DNA that activates ATR-dependent stress responses such as G2/M arrest and apoptosis [44]. To avoid these events at unrepaired CPDs, the replication mechanism uses DNA polymerases capable of bypassing CPD. In *E. coli*, the SOS response activates polV to a translesion synthesis polymerase by transferring RecA-ATP to it from a RecA filament [45]. In human cells, the *XPV* gene (defective in the xeroderma pigmentosum variant complementation group) codes for pol eta, a polymerase able to bypass CPD by incorporating A opposite a T or C in a CPD (reviewed in [46]). Correspondingly, cells from a squamous cell carcinoma from an XPV patient were found to generate recurrent chromosome abnormalities as they were passaged *in vitro*. These were dicentric chromosomes, particularly telomere–telomere bridges, indicative of telomeric damage [16]. It seems likely, then, that CPD accumulating in the telomere are especially reliant on bypass to avoid replication gaps. In the absence of bypass, these replication gaps would be frequent enough to trigger telomeric double-strand breaks and telomere–telomere bridges, the same kinds of genetic catastrophes that repair suppression aims to avoid.

## Materials and Methods

### Cells and UV Irradiation

Each experiment was performed with two different primary human fibroblast cell strains. The first strain was derived from breast reduction tissue from a healthy 25-year old female [47]. The other strain was the commercially available WI38, derived from lung tissue of a male foetus (ATCC, Manassas, VA). Cells were grown in high-glucose DMEM (Gibco Invitrogen) supplemented with 10% FBS and 1% penicillin/streptomycin. Cells were UV-irradiated at room temperature after replacing the medium with cold sterile phosphate buffered saline (PBS). The two cell strains have different UVC sensitivities (Figure S4). The UVC source was a germicidal lamp emitting at 254 nm. Using UVC rather than UVB avoids potential complications from photosensitized oxygen radical formation. For the telomere shortening experiment, UVB was used to maximize the likehood of telomere shortening; the source consisted of three fluorescent tubes (FS20T12/UVB/BP, Philips) filtered through a sheet of cellulose acetate to eliminate wavelengths below 290 nm (Kodacel TA-407 clear, 0.015 inch thickness; Eastman-Kodak Co.). Dose rate was measured prior to each experiment using a UVX UV-meter (UV Products, Upland, CA).

### IPoD

Purification of the DNA was performed using DNeasy Tissue Kit (Qiagen, Valencia, CA), according to the manufacturer's protocol. Purified DNA was sonicated to 500–1000 bp fragments (Branson sonifier 250, microtip, at 30% power, 3×15 sec on ice), precipitated with NaCl/ethanol, and resuspended in resuspension buffer (0.01% SDS, 1.1% Triton X 100, 1.2 mM EDTA, 16.7 mM Tris-Cl pH 8.1, 167 mM NaCl). DNA was denatured by boiling 10 min, incubated with the CPD-specific antibody (D194-1, MBL, Woburn, MA) [48] overnight at 4°C and then with a rabbit anti-mouse secondary antibody for 1 hour. The anti-CPD antibody was used in molar excess to CPD to ensure that each damaged dipyrimidine was pulled down regardless of its local sequence or slight variations in the binding affinity of the antibody to each dipyrimidine type. Molar excess is indicated by the linearity of the dose-response with respect to substrate (Figure 1B). Antibody-bound DNA was pulled down using Staph A beads (Calbiochem). The bead/DNA complexes were washed 2 times with wash buffer 1 (2 mM EDTA, 50 mM Tris-Cl pH 8.0) and 4 times with wash buffer 2 (100 mM Tris-Cl pH 8.0, 500 mM LiCl, 1% NP40, 1% deoxycholic acid). DNA was eluted from the staph A beads with elution buffer (50 mM NaHCO_3_, 1% SDS) and the eluted DNA was cleaned using a Qiagen PCR purification kit to remove salts and SDS prior to PCR.

In the indicated experiments, CPD were removed before the PCR reaction (but after the IP step) using cloned *E. coli* CPD photolyase (kindly provided by Drs. C. Selby and A. Sancar). The CPD photoreactivation mix (10 mM Tris-HCl pH 7.6, 10 mM NaCl, 2 mM EDTA, 20 mM DTT, 0.2 mg/mL BSA, 0.1 µL CPD photolyase) was added to the DNA and exposed for 1 h to UVA light from eight F20T12BL lamps (Spectra Mini, Daavlin Co., Bryan, OH) passed through filters to remove UVB and UVC. The DNA was then cleaned using a PCR purification kit (Qiagen).

For PCR reactions, 20 cycles of amplification were performed on a Biometra TGradient thermal cycler with Taq polymerase in 10 mM Tris/HCl, 1.5 mM MgCl_2_, 50 mM KCl, pH 8.3 and 200 µM each dNTP (Roche Molecular Biochemicals, Indianapolis, IN). A test run of PCR using different amounts of starting material was done on each sample and on each primer set to ensure the amplification lay in the exponential portion of the amplification reaction. The following primers were used: For the telomere sequence: 5′GGTTTTTGAGGGTGAGGGTGAGGGTGAGGGTGAGGGT and 5′TCCCGACTATCCCTATCCCTATCCCTATCCCTATCCCTA [19]. The underlined bases are mismatched with respect to the telomere sequence. For the *p53* gene: 5′CTGCCTCTTGCTTCTCTTTTCC and 5′GGTTTCTTCTTTGGCTGGG, giving a PCR product of 309 bp. For 28S ribosomal DNA: 5′GTAGAATAAGTGGGAGGCCCCCGG and 5′AGGCCCCGCTTTCACGGTCTGTATTCG, giving a PCR product of 368 bp. For the *CYTB* gene in mitochondrial DNA: 5′CCCTAGCCAACCCCTTAAAC and 5′TTGGCTTAGTGGGCGAAATA, giving a PCR product of 297 bp. The agarose gel was scanned and quantification was done using ImageQuant 5.0 software (Molecular Dynamics). For *p53*, 28S and mtDNA, the band was simply quantified and the background was subtracted from the signal. For the telomere sequence, the PCR primers can anneal varying distances apart on the telomeric repeat, so the PCR product is not a single-size product but rather an assortment of DNA fragments over a size range. We therefore ran telomeric samples on the agarose gel for a few minutes (to let the DNA enter the gel and to separate the PCR product from the primers), making the smear band-like. The entire smear was quantified using the same technique as for coding regions.

### DNA Damage Immunoblot

Oligonucleotides used in dot-blot experiments are depicted in Table 1. 400 ng of each double-strand oligo was irradiated with the indicated UVC doses using a 254 nm source. The irradiated DNA was denatured and applied onto a nitrocellulose membrane using a dot-blot apparatus. CPD-containing DNA on the membrane was visualized using a CPD-specific antibody (D194-1, MBL, Woburn, MA) [48] followed by a secondary anti-mouse-HRP antibody (Santa Cruz Biotechnology, Santa Cruz CA) and revealed by chemiluminescence (Denville, Metuchen, NJ). Different film exposures were scanned and quantification was done using ImageQuant 5.0 software (Molecular Dynamics).

**Table 1:** List of oligonucleotides used for the dot-blot experiment.

Oligo Name	Sequence (5′→3′)
Telomere	*X*(CCCTAA)_10_ *Y*
Repeat	*X*(CCTGAA)_10_ *Y*
Equi-diPyr #1	*X* CCTGACTAGTCGAAATCTCCTCGGACCGAAGAGCTTTGAGGTCCCTGATTGAGCCTGGAA *Y*
Equi diPyr #2	*X* CCCTTCGTTGGAGTCCCTTTTCCGGGTGGCCTCCAATCCCCATCCTTTGGCTGTTTGGCC *Y*
No-diPyr	*X* CATGTGTGTGCGTATACACGTGCGTACGTATACATATGTGTACGCAGATGCAGCGTGATA *Y*
Telo 12	*X* CATGTGTGTGCGTATACACGTGCG(CCCTAA)_2_TGTGTACGCAGATGCAGCGTGATA *Y*
Equi 12	*X* CATGTGTGTGCGTATACACGTGCGCCCAAAGGCCAGTGTGTACGCAGATGCAGCGTGATA *Y*
Telo 24	*X* CATGTGTGTGCGTATACA(CCCTAA)_4_CGCAGATGCAGCGTGATA *Y*
Equi 24	*X* CATGTGTGTGCGTATACATGCCCCCGGACCAAAGGGCTTTGACGCAGATGCAGCGTGATA *Y*
Telo 30	*X* CATGTGTGTGCGTATACA(CCCTAA)_5_TGCAGCGTGATA *Y*
Telo 36	*X* CATGTGTGTGCG(CCCTAA)_6_TGCAGCGTGATA *Y*
Equi 36	*X* CATGTGTGTGCGGTAACACCTTGGGCCGAAGGGCTTTGGGGTCCCTAATGCAGCGTGATA *Y*
Telo 42	*X* CATGTGTGTGCG(CCCTAA)_7_GTGATA *Y*
Telo 48	*X* CATGTG(CCCTAA)_8_GTGATA *Y*
Equi 48	*X* CATGTGTAGCGGAAACCCCCCCGGACCAAAGGGCTTTGAGGTCCCTAATTGGGCGTGATA *Y*
Telo 60	*X*(CCCTAA)_10_ *Y*
Equi 60	*X* CCCAATTAGCCAAAATCCCCCCGGACCAAGGAACCTTGGGGTCCCTGGTTGGGCCTGGAA *Y*
*X*	GTATACGCGTATGCATATGCA
*Y*	TGTGCATATGCACACGTGTGC

Even where only one of the two strands is listed in the table, each oligonucleotide was double-stranded when irradiated. The sequences X and Y at each end of the single-strand oligos were used generate the complementary strand by PCR amplification. It is important to note that there is no dipyrimidine site in the X and Y sequences, so those regions cannot generate cyclobutane pyrimidine dimers.

### Telomere Shortening Experiment

Cells were irradiated with different UVB doses (0, 10, 50, 100 and 200 J/m^2^). After the irradiation, cells from each condition were allow to grow until they reached full confluency. When cells from every exposure condition reached 100% confluency, they were all passaged 1∶4. This precaution was taken to assure that UV-irradiated cells did not undergo fewer population doublings than unirradiated ones at the same passage number. (A disadvantage of this design is that mortality at the higher UV doses would cause more divisions of the remaining living cells to compensate, possibly leading to faster telomere shortening at these doses. However, because telomere shortening was not seen, this absence is conclusive.) UV-induced telomere shortening would be obscured if UV also reduced the number of cell doublings by decreasing the cell density at confluence. This effect would reduce the extent of normal, replication-related, telomere shortening. The cell density reduction apparently did not occur here. Because each cell lineage was split at the same ratio, a 25% reduction in cell density of treated cells compared to untreated would result in a (0.75)^16^ = 100-fold difference in cell number after 16 passages (from X12 to X28). But no difference in the final amount of DNA harvested was observed between any of the UV doses.

Terminal restriction fragment length measurements were obtained using the Telo TTAGGG telomere length assay kit (Roche Molecular Biochemicals, Indianapolis, IN) as done previously [47]. Briefly, 2 mg of HinfI/RsaI-digested genomic DNA were separated on 0.8% agarose gels and Southern blotted onto a Hybond-N+ nylon membrane (Amersham Biosciences, Piscataway, NJ). After UV-fixation of DNA fragments onto the membrane, membranes were hybridized with digoxigenin-labeled telomere-specific probe (TTAGGG)4. After washing out non-bound probe, membranes were incubated with a digoxigenin-specific antibody covalently coupled to alkaline phosphatase. Finally, the telomere fragments were visualized by a chemiluminescent substrate (CDP-star, Roche Molecular Biochemicals, Indianapolis, IN). TRF lengths were determined by comparing the signals relative to a standard molecular weight using ImageQuant 5.0 software (Molecular Dynamics). All lanes were divided into 75 intervals, and the mean TRF length was defined as S(ODi)/S(ODi/Li), in which ODi is the chemiluminescent signal and Li is the length of the TRF fragment at position I [49]. Although TRF fragments have one terminus in the pre-telomeric region, changes in TRF length reflect changes in telomere length.

## Supporting Information

Figure S1 Specificity of IPoD for UV-induced cyclobutane pyrimidine dimers. Human diploid fibroblasts were irradiated with 20 J/m^2^ UVC and DNA fragments were then incubated with antibody to CPD, antibody to Bcl xL protein, or without Ab. After immunoprecipitation, the pulldown and supernatant were amplified with telomere-specific PCR primers and subjected to agarose gel electrophoresis.
(1.59 MB EPS)

Figure S2 Linearity of the IPoD to up to 30 J/m^2^ UVC. Human diploid fibroblasts were irradiated with the indicated UVC dose and CPD-containing DNA was immunoprecipitated using the IPoD technique. After immunoprecipitation, the pulldown and supernatant DNA were amplified with telomere PCR primers and subjected to agarose gel electrophoresis.
(5.29 MB EPS)

Figure S3 Susceptibility of telomere sequence to DNA fragmentation and enzymatic treatment. (A) Fragmentation by sonication for telomeric DNA repeats is the same as for a coding region. Total genomic DNA was sonicated to obtain fragments size between 0.5 and 1 kb. DNA was separated on a native agarose gel and transfered onto a nylon membrane by capillary action. Digoxigenin-labeled DNA probe specific for the telomere sequence or *p53* was then hybridized to the membrane and anti-digoxigenin coupled to alkaline phosphatase was used to reveal sequence specific signal. This figure shows that the sonication efficiency is not influenced by the repeat nature of the telomere sequence. (B) Accessibility to enzymes is similar in the telomeric region and a coding region. DNA from human cells was irradiated or not with 20 J/m^2^ UVC. DNA was then treated or not with CPD photolyase to remove CPD followed by a treatment using T4 Endonuclease V to convert CPD into single-strand breaks. Treated DNA was subjected to a denaturing alkaline agarose gel, transferred to a nylon membrane, and hybridized with a telomere- or *p53*-specific probe. This figure shows that photolyase is able to repair all CPD present in both a coding region (*p53*) and repeated DNA (telomere). The far left and right lanes are molecular weight markers.
(27.04 MB EPS)

Figure S4 UV sensitivity of primary diploid human skin fibroblasts and WI38 primary human lung fibroblasts. Cells were irradiated with the indicated UVC dose (0 to 100 J/m^2^) and the survival was evaluated 24 h post-irradiation using trypan blue. The result depicted in this graph is derived from triplicate experiments.
(0.06 MB DOC)

## References

[1] Osterhage JL, Friedman KL (2009). Chromosome end maintenance by telomerase.. J Biol Chem.

[2] Henle ES, Han Z, Tang N, Rai P, Luo Y, Linn S (1999). Sequence-specific DNA cleavage by Fe2+-mediated fenton reactions has possible biological implications.. J Biol Chem.

[3] Oikawa S, Tada-Oikawa S, Kawanishi S (2001). Site-specific DNA damage at the GGG sequence by UVA involves acceleration of telomere shortening.. Biochemistry.

[4] Petersen S, Saretzki G, von Zglinicki T (1998). Preferential accumulation of single-stranded regions in telomeres of human fibroblasts.. Exp Cell Res.

[5] Sitte N, Saretzki G, von Zglinicki T (1998). Accelerated telomere shortening in fibroblasts after extended periods of confluency.. Free Radic Biol Med.

[6] von Zglinicki T, Saretzki G, Docke W, Lotze C (1995). Mild hyperoxia shortens telomeres and inhibits proliferation of fibroblasts: a model for senescence?. Exp Cell Res.

[7] Cadet J, Sage E, Douki T (2005). Ultraviolet radiation-mediated damage to cellular DNA.. Mutat Res.

[8] Douki T, Reynaud-Angelin A, Cadet J, Sage E (2003). Bipyrimidine photoproducts rather than oxidative lesions are the main type of DNA damage involved in the genotoxic effect of solar UVA radiation.. Biochemistry.

[9] Wang SY (1976). Photochemistry and Photobiology of Nucleic Acids..

[10] Brunk CF (1973). Distribution of dimers in ultraviolet-irradiated DNA.. Nat New Biol.

[11] Gueron M, Shulman RG (1968). Energy transfer in polynucleotides.. Annu Rev Biochem.

[12] Nordlund TM (2007). Sequence, structure and energy transfer in DNA.. Photochem Photobiol.

[13] Lam LH, Reynolds RJ (1987). DNA sequence dependence of closely opposed cyclobutyl pyrimidine dimers induced by UV radiation.. Mutat Res.

[14] Sedgwick SG (1976). Misrepair of overlapping daughter strand gaps as a possible mechanism for UV induced mutagenesis in UVR strains of Escherichia coli: a general model for induced mutagenesis by misrepair (SOS repair) of closely spaced DNA lesions.. Mutat Res.

[15] Witkin EM (1976). Ultraviolet mutagenesis and inducible DNA repair in Escherichia coli.. Bacteriol Rev.

[16] Aledo R, Aurias A, Avril MF, Dutrillaux B (1989). Jumping end-to-end dicentrics in a case of squamous cell carcinoma from a patient with xeroderma pigmentosum.. Cancer Genet Cytogenet.

[17] Munoz P, Blanco R, Flores JM, Blasco MA (2005). XPF nuclease-dependent telomere loss and increased DNA damage in mice overexpressing TRF2 result in premature aging and cancer.. Nat Genet.

[18] Tornaletti S, Pfeifer GP, Pfeifer GP (1996). Ligation-Mediated PCR for Analysis of UV damage.. Technologies for detection of DNA damage and mutations.

[19] Cawthon RM (2002). Telomere measurement by quantitative PCR.. Nucleic Acids Res.

[20] Kruk PA, Rampino NJ, Bohr VA (1995). DNA damage and repair in telomeres: relation to aging.. Proc Natl Acad Sci U S A.

[21] Saldanha SN, Andrews LG, Tollefsbol TO (2003). Assessment of telomere length and factors that contribute to its stability.. Eur J Biochem.

[22] Surralles J, Ramirez MJ, Marcos R, Natarajan AT, Mullenders LH (2002). Clusters of transcription-coupled repair in the human genome.. Proc Natl Acad Sci U S A.

[23] Oberley MJ, Farnham PJ (2003). Probing chromatin immunoprecipitates with CpG-island microarrays to identify genomic sites occupied by DNA-binding proteins.. Methods Enzymol.

[24] Smith CA, Baeten J, Taylor JS (1998). The ability of a variety of polymerases to synthesize past site-specific cis-syn, trans-syn-II, (6-4), and Dewar photoproducts of thymidylyl-(3′-->5′)-thymidine.. J Biol Chem.

[25] Rochette PJ, Bastien N, McKay BC, Therrien JP, Drobetsky EA, Drouin R (2002). Human cells bearing homozygous mutations in the DNA mismatch repair genes hMLH1 or hMSH2 are fully proficient in transcription-coupled nucleotide excision repair.. Oncogene.

[26] Balajee AS, May A, Bohr VA (1999). DNA repair of pyrimidine dimers and 6-4 photoproducts in the ribosomal DNA.. Nucleic Acids Res.

[27] Christians FC, Hanawalt PC (1993). Lack of transcription-coupled repair in mammalian ribosomal RNA genes.. Biochemistry.

[28] Stevnsner T, May A, Petersen LN, Larminat F, Pirsel M, Bohr VA (1993). Repair of ribosomal RNA genes in hamster cells after UV irradiation, or treatment with cisplatin or alkylating agents.. Carcinogenesis.

[29] Clayton DA, Doda JN, Friedberg EC (1974). The absence of a pyrimidine dimer repair mechanism in mammalian mitochondria.. Proc Natl Acad Sci U S A.

[30] LeDoux SP, Wilson GL, Beecham EJ, Stevnsner T, Wassermann K, Bohr VA (1992). Repair of mitochondrial DNA after various types of DNA damage in Chinese hamster ovary cells.. Carcinogenesis.

[31] Pascucci B, Versteegh A, van Hoffen A, van Zeeland AA, Mullenders LH, Dogliotti E (1997). DNA repair of UV photoproducts and mutagenesis in human mitochondrial DNA.. J Mol Biol.

[32] Batista LF, Kaina B, Meneghini R, Menck CF (2009). How DNA lesions are turned into powerful killing structures: insights from UV-induced apoptosis.. Mutat Res.

[33] Paulsen RD, Cimprich KA (2007). The ATR pathway: fine-tuning the fork.. DNA Repair (Amst).

[34] Opresko PL, Fan J, Danzy S, Wilson DM, Bohr VA (2005). Oxidative damage in telomeric DNA disrupts recognition by TRF1 and TRF2.. Nucleic Acids Res.

[35] Oexle K (1998). Telomere length distribution and Southern blot analysis.. J Theor Biol.

[36] Vorlickova M, Kejnovska I, Tumova M, Kypr J (2001). Conformational properties of DNA fragments containing GAC trinucleotide repeats associated with skeletal displasias.. Eur Biophys J.

[37] Vorlickova M, Chladkova J, Kejnovska I, Fialova M, Kypr J (2005). Guanine tetraplex topology of human telomere DNA is governed by the number of (TTAGGG) repeats.. Nucleic Acids Res.

[38] Livingstone-Zatchej M, Marcionelli R, Moller K, de Pril R, Thoma F (2003). Repair of UV lesions in silenced chromatin provides in vivo evidence for a compact chromatin structure.. J Biol Chem.

[39] Schnell JR, Berman J, Bloomfield VA (1998). Insertion of telomere repeat sequence decreases plasmid DNA condensation by cobalt (III) hexaammine.. Biophys J.

[40] Denchi EL (2009). Give me a break: How telomeres suppress the DNA damage response.. DNA Repair (Amst).

[41] Ward JF (1994). The complexity of DNA damage: relevance to biological consequences.. Int J Radiat Biol.

[42] Lopes M, Foiani M, Sogo JM (2006). Multiple mechanisms control chromosome integrity after replication fork uncoupling and restart at irreparable UV lesions.. Mol Cell.

[43] Heller RC, Marians KJ (2006). Replication fork reactivation downstream of a blocked nascent leading strand.. Nature.

[44] Myers K, Gagou ME, Zuazua-Villar P, Rodriguez R, Meuth M (2009). ATR and Chk1 suppress a caspase-3-dependent apoptotic response following DNA replication stress.. PLoS Genet.

[45] Jiang Q, Karata K, Woodgate R, Cox MM, Goodman MF (2009). The active form of DNA polymerase V is UmuD'2C-RecA-ATP.. Nature.

[46] Cordonnier AM, Fuchs RP (1999). Replication of damaged DNA: molecular defect in xeroderma pigmentosum variant cells.. Mutat Res.

[47] Rochette PJ, Brash DE (2008). Progressive apoptosis resistance prior to senescence and control by the anti-apoptotic protein BCL-xL.. Mech Ageing Dev.

[48] Mori T, Nakane M, Hattori T, Matsunaga T, Ihara M, Nikaido O (1991). Simultaneous establishment of monoclonal antibodies specific for either cyclobutane pyrimidine dimer or (6-4)photoproduct from the same mouse immunized with ultraviolet-irradiated DNA.. Photochem Photobiol.

[49] de Lange T, Shiue L, Myers RM, Cox DR, Naylor SL, Killery AM, Varmus HE (1990). Structure and variability of human chromosome ends.. Mol Cell Biol.

