# Supplementary figures and images for: Human Telomeres Are Hypersensitive to UV-Induced DNA Damage and Refractory to Repair
Source: PLoS Genet. 2010 Apr 29;6(4):e1000926. doi: 10.1371/journal.pgen.1000926 (PMC2861706; doi:10.1371/journal.pgen.1000926)

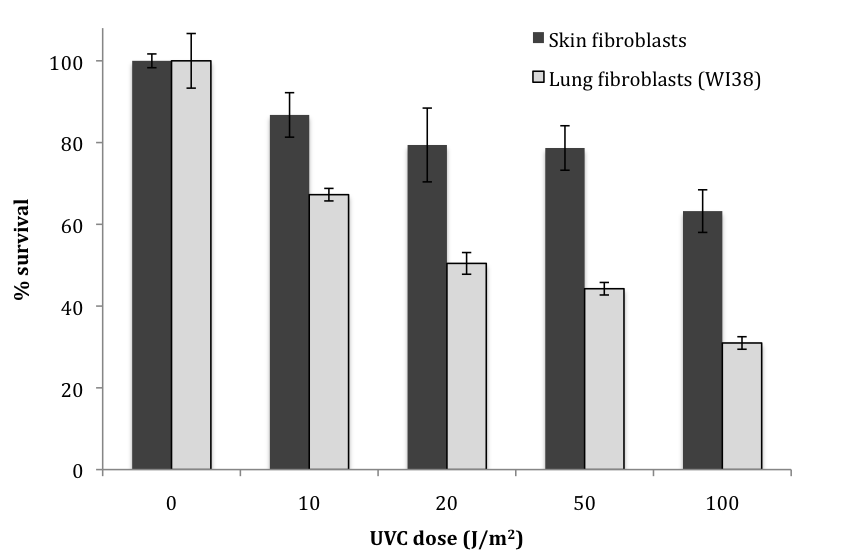

Supplement: Figure S4 — UV sensitivity of primary diploid human skin fibroblasts and WI38 primary human lung fibroblasts. Cells were irradiated with the indicated UVC dose (0 to 100 J/m2) and the survival was evaluated 24 h post-irradiation using trypan blue. The result depicted in this graph is derived from triplicate experiments. (0.06 MB DOC) [file pgen.1000926.s004.doc]
